# Rates of buprenorphine prescribing and racial disparities among patients with opioid overdose

**DOI:** 10.1016/j.dadr.2024.100298

**Published:** 2024-11-04

**Authors:** Kimberly Y. Chieh, Lauren A. Walter, Karen L. Cropsey, Li Li

**Affiliations:** a Department of Psychiatry and Behavioral Neurobiology, University of Alabama at Birmingham, Birmingham, AL 35294, United States; b Department of Emergency Medicine, University of Alabama at Birmingham, Birmingham, AL 35294, United States

**Keywords:** Overdose, Buprenorphine, Prescription, Disparities, Opioid use disorder

## Abstract

**Background:**

Awareness of the relationship between real-world buprenorphine prescribing and overdose frequency is limited, especially in the Southeastern United States. We described buprenorphine prescribing rates for patients experiencing nonfatal opioid overdoses in the context of overdose frequency.

**Methods:**

Electronic medical records review was conducted at an urban, academic hospital in Alabama from January 1 through December 31, 2021. Patients with opioid use disorder (OUD) and nonfatal opioid overdoses, dispositioned from either the emergency department (ED), inpatient, or outpatient affiliated clinics, were identified by International Classification of Diseases-10 codes.

**Results:**

The study included 358 unique patients. Many patients were white (71.5 %), male (59.2 %), and uninsured (54.2 %), with a mean age of 42.0±12.8 years. The majority (85.5 %) experienced one to three overdoses, and 14.5 % of patients had more than three overdoses. The buprenorphine prescription rate increased to 55.8 % when patients had more than three overdoses, compared to one overdose (34.5 %) and two to three overdoses (37.4 %) (*p*=0.025). Compared to females, more males overdosed more than once (*p*=0.004). Black patients were less likely to receive buprenorphine prescriptions than white patients (27.3 % vs. 44.5 %, *p*=0.004). Compared to patients with multiple overdoses, more patients with one overdose had public insurance (*p*=0.028) and were less likely to present to the ED (*p*<0.001).

**Conclusion:**

Under-prescribing of buprenorphine is high among patients with OUD and opioid overdoses, even in patients with multiple overdoses, and there appear to be racial disparities in prescribing. Our findings indicate clinical opportunities for improving buprenorphine prescribing and reducing the current disparities.

## Highlights

• Most patients had 1–3 nonfatal opioid overdoses within the 1-year study period.
• Males overdosed more frequently than females.
• Buprenorphine is under-prescribed among patients with nonfatal opioid overdoses.
• Buprenorphine is prescribed less in patients with ≤3 overdoses versus >3 overdoses.
• Black patients are less likely to receive buprenorphine than white patients.

## 1 Introduction

Worldwide, both nonfatal and fatal opioid-related overdoses are a significant morbidity and mortality. The World Health Organization (WHO) estimates that of the 600,000 worldwide deaths attributable to drug use in 2019, almost 80 % were related to opioids, and approximately 125,000 of these deaths were from an opioid overdose. In recent years, more overdoses have occurred in several countries due to increasing reliance on opioids for chronic pain management and increasing availability and use of illicit opioids (Ju et al., 2022). In 2021, the WHO reports about 60 million people used opioids across the globe, and of the 39.5 million people with drug use disorders, only 1 in 5 were receiving treatment for their drug use. Only half of countries have access to effective treatment options, and less than 10 % of people worldwide with opioid dependence are actually receiving treatment (World Health Organization., 2023).

In the United States, the ongoing opioid crisis has also contributed to increased numbers of nonfatal and fatal opioid-related overdoses, and the crisis has only been exacerbated by the COVID-19 pandemic. Per the Centers for Disease Control and Prevention, approximately 107,622 deaths were caused by drug overdoses in 2021. Among these deaths, 80,816 were related to opioids (Centers for Disease Control and Prevention, 2022a, Centers for Disease Control and Prevention, 2022b). The number of total opioid overdoses also increased by approximately 37 % from 2019 to 2021 (Centers for Disease Control and Prevention, 2022a, Centers for Disease Control and Prevention, 2022b). In addition, previous studies have reported increased emergency department (ED) visits following nonfatal opioid overdoses during 2020 compared with previous years (Ochalek et al., 2020, Patel et al., 2021, Soares et al., 2022). Nonfatal opioid overdoses are a strong predictor of subsequent fatal overdose and increased short-term mortality (Caudarella et al., 2016). For example, from 2011 to 2015, among persons in Massachusetts who had a nonfatal overdose, 6.2 % died of an opioid-related overdose within 1 year and 9.3 % within 2 years (Massachusetts Department of Public Health., 2017). After a nonfatal opioid overdose, treatment with methadone or buprenorphine has been found to reduce opioid-related mortality by 59 % and 38 %, respectively (Larochelle et al., 2018). Despite this, studies report that only a minority of patients treated in EDs for nonfatal opioid overdoses subsequently receive one of the three Food and Drug Administration approved medications for opioid use disorder (MOUD), ranging from less than 5 % of Medicaid patients to 11.1 % of commercially insured patients (Frazier et al., 2017, Kilaru et al., 2020, Koyawala et al., 2019). Thus, lack of utilization of evidence-based treatment options remains a prominent issue in patients with nonfatal opioid overdoses.

Despite robust evidence supporting the effectiveness of buprenorphine treatment in reducing the risk of nonfatal opioid overdose events and deaths, buprenorphine initiation and retention remains a challenge. A nationwide study estimated that only 15 % of patients who had opioid use disorder (OUD) upon being admitted to Veterans Health Administration hospitals received any MOUD, and initiation of MOUD treatment plus linkage to post discharge care was provided in less than 2 % of cases (Priest et al., 2020). More challengingly, adherence to buprenorphine is low (Samples et al., 2018, Timko et al., 2016). For example, one study showed that in patients with OUD, 10 % discontinued buprenorphine in the first week, over 25 % discontinued in the first month and nearly two-thirds discontinued in the first six months following buprenorphine initiation (Samples et al., 2018). This study also found that risk factors for buprenorphine discontinuation included low initial buprenorphine dosage, male sex, minority race/ethnicity including Black and Hispanic patients, comorbid substance use disorders, and opioid overdose history, among other factors (Samples et al., 2018). Racial disparities in treatment and opioid-related outcomes have worsened since the COVID pandemic (Schiff et al., 2021).

In Alabama, a state in the Southeastern region of the United States, most reported fatal drug overdose deaths have involved opioids (Alabama Department of Public Health, 2019). From 2019–2020, drug overdoses in Alabama increased by 20 % (Alabama Department of Mental Health, 2021). Jefferson County, the most populous county in Alabama, saw a 32.5 % increase in fatal opioid overdoses in the first six months of 2020 (Yurkanin., 2020). Prior studies have also shown disparities in opioid overdose outcomes, demonstrating a dramatic increase in the number of Black patients impacted by opioid overdose deaths, as compared to white patients, with the largest discrepancies in the Southern states (Patel et al., 2021, Townsend et al., 2022). Furthermore, consideration of buprenorphine prescribing also demonstrates disparities, with fewer Black patients receiving this MOUD (Lagisetty et al., 2019, Schuler et al., 2021). However, little data exist on actual buprenorphine prescribing patterns and potential disparities among patients with OUD who have experienced a nonfatal overdose, especially in the Southeastern United States, as it relates to overdose frequency. Thus, this study aimed to determine: 1) buprenorphine prescribing patterns among patients with OUD and nonfatal opioid overdoses; 2) the relationship between buprenorphine prescribing and the frequency of nonfatal overdoses; and 3) the sociodemographic characteristics and health encounter details associated with buprenorphine prescribing.

## 2 Methods

This was a retrospective and observational study approved by the University of Alabama at Birmingham (UAB) Institutional Review Board (approved 9/1/2022, approval number-300009508), and informed consent was waived. All procedures were performed in accordance with relevant laws and institutional guidelines.

UAB Hospital is an urban, academic, tertiary care hospital with over 1100 inpatient beds and an annual ED volume of greater than 75,000 patient visits. Patients who were diagnosed with OUD and nonfatal opioid-related overdose at the UAB Hospital from January 1, 2021, to December 31, 2021, were identified by electronic medical records (EMR). EMR search was based on patients who were diagnosed with OUD per the International Classification of Diseases-10 (ICD-10) code criteria and presented in the inpatient, outpatient, and ED settings with poisoning due to any form of opioid per T40.0-T40.6 and excluding T40.5 per the ICD-10 code criteria. Two researchers (KC and LL) independently conducted EMR reviews to abstract patient data for the 1-year study period. We received aggregate deidentified data and assessed for duplicate entries to identify 358 unique patients. We then identified specific demographic characteristics and presence of buprenorphine prescription for these 358 patients. Also, these same two researchers independently conducted data analyses in order to ensure data was processed appropriately, and the same statistical results were obtained.

Distinct hospital locales considered included the ED, the inpatient setting, as well as the hospital-affiliated outpatient clinical settings that are part of the hospital service. ‘Encounter’ was classified by the dispositioning locale. Demographic characteristics, including sex, age, race/ethnicity, encounter setting, presence of other comorbidities, and registration verified insurance status, were obtained from the EMR. Type of opioid overdose (e.g. fentanyl, heroin) was also noted when available. Comorbidities include psychiatric disorders (all F codes except F10–19) and all other substance use disorders except OUD (F11). Buprenorphine prescriptions, as documented in the EMR, and frequency of overdose presentations per patient during the study period were also considered. However, our EMR could not verify if buprenorphine prescriptions were dispensed in a pharmacy as prescribed. Number of overdoses was defined as the number of patient presentations due to opioid overdose within the study period. Overdose frequency was categorized as one overdose, two to three overdoses, or more than three overdoses. Descriptive statistical and Chi-square analyses were performed using SPSS version 27 (IBM), and post-hoc analyses including Bonferroni Adjustment were performed using Microsoft Excel.

## 3 Results

### 3.1 Patient characteristics

The study included 358 unique patients who presented to the UAB Hospital with OUD and nonfatal opioid-related overdoses in 2021 (Table 1). The average age for patients was 42.0 (SD=12.8 years). Many patients were white (71.5 %), male (59.2 %), and non-Hispanic or Latino (96.0 %). The majority were uninsured (54.2 %) or had public health insurance (29.6 %), and the remaining patients (16.2 %) had private insurance.

**Table 1:** Demographics of patients in 2021.

**Demographics**	**Patients with non-fatal opioid overdoses (n = 358)**
Sex	
Male Female	212 (59.2 %) 146 (40.8 %)
Race^a^	
White Black	256 (71.5 %) 88 (24.6 %)
Comorbidities Present	213 (59.5 %)
Insurance Status	
Uninsured Public Private	194 (54.2 %) 106 (29.6 %) 58 (16.2 %)
Visit/Disposition Location^b^	
Emergency Department Inpatient Outpatient	172 (48.1 %) 79 (22.1 %) 99 (27.2 %)

Note: Data are presented in number and percentage, N (%). Percentage is out of

each subcategory. ^a^: Numbers do not add to 100 % in each group due to missing

data. ^b^: Numbers do not add to 100 % in each group due to missing data.

### 3.2 Nonfatal opioid-related overdoses and frequency

During the 1-year study period, the majority (n=306, 85.5 %) of patients overdosed one to three times; 116 (32.4 %) patients once, 80 (22.3 %) patients twice, and 110 (30.7 %) patients three times. Fifty-two patients (14.5 %) overdosed more than three times, and one patient presented to UAB Hospital a total of 13 times in 2021, each presentation following a nonfatal opioid overdose. Seventy (19.6 %) patients primarily overdosed on heroin, 89 (24.9 %) overdosed on fentanyl, and 9 (2.5 %) patients overdosed on methadone.

All three overdose frequency groups consisted of a majority of white male patients (Table 2). There were no differences in overdose rates by race across frequency groups. The ED saw the majority of all overdoses and was more likely to care for those with repeat overdoses than both the inpatient and outpatient settings, *X*^2^ (6, *N* = 358) = 24.397, *p* < 0.001). Additionally, a sex-specific pattern for overdose frequency was observed in which females were more likely to have a single overdose, while male patients were more likely to have multiple overdoses, *X*^2^ (2, *N* = 358) = 10.982, *p* = 0.004. In general, patients who were uninsured or publicly insured made up the majority of all overdoses during the study period. Compared to patients with multiple overdoses, more patients with one overdose had public insurance during the study period, *X*^2^ (4, *N* = 358) = 10.861, *p* = 0.028.

**Table 2:** Characteristics of patients split into groups by overdose frequency in 2021.

		**1 overdose** (N=116)	**2–3 overdoses** (N=190)	**>3 overdoses** (N=52)	***p*****-value**
**Age, years (SD)**		40.3±18.8	38.5±14.4	41.2±13.5	0.390
**Buprenorphine prescribing**		40 (34.5 %)	71 (37.4 %)	29 (55.8 %)	**0.025**
**Sex**	Female	61 (52.6 %)	70 (36.8 %)	15 (28.8 %)	**0.004**
	Male	55 (47.4 %)	120 (63.2 %)	37 (71.2 %)	
**Encounter**^**a**^	Emergency	36 (31.0 %)	108 (56.8 %)	28 (53.8 %)	**0.001**
	Inpatient	32 (27.6 %)	32 (16.8 %)	15 (28.8 %)	
	Outpatient	45 (38.8 %)	46 (24.2 %)	8 (15.4 %)	
**Race**^**b**^	White	81 (68.9 %)	136 (71.6 %)	39 (75.0 %)	0.885
	Black	30 (25.9 %)	46 (24.2 %)	12 (23.0 %)	
**Comorbidities**	Present	71 (61.2 %)	113 (59.5 %)	29 (55.8 %)	0.802
**Insurance**	Uninsured	55 (47.4 %)	105 (55.2 %)	34 (65.4 %)	**0.028**
	Public	46 (39.7 %)	48 (25.3 %)	12 (23.1 %)	
	Private	15 (12.9 %)	37 (19.5 %)	6 (11.5 %)	

Note: Data are presented in number and percentage, N (%). Percentage is out of each overdose frequency group. ^a^: Numbers do not add to 100 % in each group due to missing data (3 in the 1 overdose group, 4 in the 2–3 overdoses group, and 1 in the group of >3 overdoses). ^b^: Numbers do not add to 100 % in each group due to missing data (5 in the 1 overdose group, 8 in the 2–3 overdoses group, and 1 in the group of >3 overdoses). *P*-values are from Chi-square analysis and compare all three groups.

### 3.3 Buprenorphine prescribing and racial disparities

Among the 358 patients, 218 (60.8 %) did not receive a buprenorphine prescription despite experiencing at least one nonfatal overdose during the study period; however, patients experiencing more than three overdoses were more likely to receive buprenorphine (55.8 %; *X*^*2*^ *(2, N = 358) = 7.345, p = 0.025*). Black patients were significantly less likely to receive a prescription for buprenorphine than white patients regardless of overdose frequency, *X*^2^ (1, *N* = 344) = 8.120, *p* = 0.004. Out of the 88 total Black patients, only 27.3 % (n=24) received a buprenorphine prescription, whereas 44.5 % (n=114 out of 256) white patients received a prescription.

### 3.4 Comorbidities

Comorbidities were common amongst overdose patients (59.5 %). When considering specific diagnoses, the majority (65.7 %) demonstrated concomitant substance use disorders, most commonly stimulant use disorder. Regarding concurrent psychiatric disorders, the rate of mood disorders (16 %) was also relatively high among overdose patients, followed by anxiety and trauma-related disorders (13 %).

## 4 Discussion

To the best of the authors’ knowledge, this is the first study to examine the association between buprenorphine prescribing and the frequency of nonfatal opioid overdoses in patients with OUD. What is perhaps most immediately notable is the sheer number of repeat overdoses experienced by patients. Over two-thirds of patients experienced more than one opioid-related overdose within the study timeframe. As an overdose is considered a life-threatening emergency with significant associated morbidity and mortality, the frequency with which persons with OUD experienced these near fatal events is an alarming finding. Further, these individuals then interacted with the healthcare community in a variety of settings, but often did not receive or were not receptive to evidence-based treatment considerations, including MOUD, which might preempt another overdose.

OUD is a treatable and chronic disorder (Taylor and Samet, 2022). If untreated, OUD is associated with significant morbidity and mortality (Strang et al., 2020). Although buprenorphine has been demonstrated to reduce fatal and nonfatal opioid overdoses, it has been underutilized (Louie et al., 2019, Peterson et al., 2020, Victor et al., 2021). Our observation of buprenorphine underutilization in patients with nonfatal opioid overdoses in this study is consistent with previous cohort studies that examined treatment patterns after a nonfatal opioid overdose. In a study among opioid overdose survivors in Pennsylvania Medicaid claims, rates of MOUD treatment increased after heroin overdose from 29.5 % to 33.0 % and after prescription opioid overdose from 13.5 % to 15.1 % (Frazier et al., 2017). A more recent study in 17,568 Massachusetts adults who survived an opioid overdose between 2012 and 2014 found that approximately 17 % received buprenorphine for a median of 4 months (Larochelle et al., 2018). In our current study, we observed that patients with multiple overdoses were more likely to be initiated on buprenorphine treatment, although reasons remain unclear. Additionally, our results showed that these patients are repeated but not efficient utilizers of the ED and medical resources (Kahler et al., 2017). Our findings echo previous reports that untreated nonfatal overdoses lead to recurrent overdoses (Goldman-Mellor et al., 2020, Guo et al., 2021, Weiner et al., 2020). Our data highlight that a history of opioid overdose is a strong risk factor for subsequent overdoses, and it is critical for patients to be treated with buprenorphine or other MOUDs when they begin to experience overdoses.

Buprenorphine prescribing has increased significantly since the start of the COVID-19 pandemic (Ali et al., 2023), however, as we described in this study, under-prescribing still persists. There are several potential reasons for this under-prescription. Our unpublished data and others show that providers may have been hesitant to apply for their X waivers or not utilizing their capacity of waivers (Ali et al., 2023). Patient stigma surrounding OUD and related medications is another barrier that prevents them from taking buprenorphine (Andraka-Christou et al., 2022, Witte et al., 2021). Other identified barriers may be related to lack of administrative support for providers, transportation access for patients, and the high cost of medical care (Godersky et al., 2019, Haffajee et al., 2018). Although our findings indicate some gaps, like buprenorphine under-prescribing, in patient care which may attribute to these barriers, our findings also suggest meaningful opportunities to improve initiation, engagement, and retention for buprenorphine treatment after a nonfatal overdose.

Regarding types of opioids involved in overdoses in this study, synthetic opioids, including illicit fentanyl, were dominant, and heroin involvement was less than 20 %, which is consistent with national trends since the COVID-19 pandemic started (May et al., 2021, Pardo et al., 2021, NEMA Research Group, 2018). Methadone contributed to less than 5 % overdose cases, which is likely due to the highly restrictive regulation of methadone prescribing in Alabama and the United States. Previous studies have also shown that people with OUD experience high rates of other concurrent substance use disorders and psychiatric disorders (Bakos-Block et al., 2020, Mahoney et al., 2021, Zhu et al., 2021). Consistent with that data, our study found that over 50 % patients with opioid overdoses had other co-occurring substance use and psychiatric disorders, especially mood disorders. Concurrent polysubstance use and psychiatric disorders are alarming because they may lead to negative treatment outcomes, including shorter retention in treatment, additional risk of repeat overdoses, and higher mortality among patients receiving MOUD (Larochelle et al., 2021, Zozula et al., 2022). Thus, providers have an important role to play in diagnosing OUD and its comorbidities, offering evidence-based buprenorphine treatment, and delivering overdose prevention to individuals who continue to use opioids and other substances. Additionally, more comprehensive assessment and treatment plans should be integrated into OUD treatment for co-occurring psychiatric disorders with fine-line therapies.

Our current and previous studies have shown that Black patients continue to face inequities in OUD treatment access, including lower rates of buprenorphine prescribing and naloxone prescribing (Patel et al., 2021, Robbins et al., 2021). Our findings and others indicate that these persistent inequities coincide with a surge in opioid overdose deaths in non-Hispanic Black patients (Larochelle et al., 2021, Patel et al., 2021). In addition to barriers stated above, structural racism may be another contributing factor to these worse outcomes and higher mortality (Williams et al., 2019). Given the health benefits of buprenorphine treatment in reducing opioid overdoses, providers need to take active steps to reduce treatment bias to improve equitable MOUD access.

Our study has several limitations. First, identification of OUD diagnosis depends on ICD-10 codes in the EMR, which were unable to be verified in each patient. Second, our findings may have limited generalizability outside the state of Alabama, a Medicaid non-expansion state, which has higher opioid-related mortality (UAB hospital catchment areas) and higher prevalence of non-insurance coverage, including public health insurance, than the United States average; these factors may translate to lower treatment rates. Additionally, due to EMR limitations we were unable to assess polysubstance use and whether buprenorphine orders were dispensed at a pharmacy. Of note, the study period was during the COVID-19 pandemic, which likely impacted patient presentations to various healthcare settings. Lastly, due to the nature of study design, the duration of each patient’s buprenorphine treatment is not known, which is a potential factor to be included in future research.

## 5 Conclusion

Our data suggest that under-prescribing of buprenorphine is high among patients with OUD and nonfatal opioid overdoses, especially among patients with three or fewer overdoses when compared to patients with more than three overdoses. Our findings also demonstrate racial disparities in buprenorphine prescribing among patients with overdoses, as disproportionately less Black patients received a buprenorphine prescription than white patients. These results demonstrate the need for buprenorphine prescribing and access to be expanded to satisfy the unmet need for appropriate treatment of those struggling with opioid overdose and OUD, with particular attention to Black patients, patients with multiple overdoses, and uninsured patients. These findings suggest meaningful opportunities to improve engagement and retention in treatment of OUD after a nonfatal overdose.

## Role of funding source

Nothing declared. This research did not receive any specific grant from funding agencies in the public, commercial, or not-for-profit sectors.

## Contributors

Kimberly Y Chieh, Lauren A Walter, Karen L Cropsey, Li Li

## CRediT authorship contribution statement

**Li Li:** Writing – original draft, Investigation, Formal analysis, Conceptualization. **Lauren A Walter:** Writing – review & editing. **Karen L Cropsey:** Writing – review & editing. **Kimberly Y Chieh:** Writing – original draft, Investigation, Formal analysis.

## Declaration of Competing Interest

The authors declare that they have no known competing financial interests or personal relationships that could have appeared to influence the work reported in this paper.
