# Supplementary material for: Rates of buprenorphine prescribing and racial disparities among patients with opioid overdose
Source: Drug Alcohol Depend Rep. 2024 Nov 4;13:100298. doi: 10.1016/j.dadr.2024.100298 (PMC11584192; doi:10.1016/j.dadr.2024.100298)
Supplement: Supplementary file 1 — Supplementary material [file mmc1.docx]

**ICD-10 codes used to identify various psychiatric comorbidities**

F10: Alcohol related disorders

F12: Cannabis related disorders

F13: Sedative, hypnotic, or anxiolytic related disorders

F14: Cocaine related disorders

F15: Mental and behavioral disorders due to use of other stimulants, including caffeine

F17: Mental and behavioral disorders due to use of tobacco

F19: Mental and behavioral disorders due to multiple drug use and use of other psychoactive substances

F20: Schizophrenia

F23: Acute and transient psychotic disorders

F25: Schizoaffective disorders

F29: Unspecified nonorganic psychosis

F31: Bipolar affective disorder

F32: Depressive episode

F33: Recurrent depressive disorder

F34: Persistent mood [affective] disorder

F39: Unspecified mood [affective] disorder

F41: Other anxiety disorders

F42: Obsessive-compulsive disorder

F43: Reaction to severe stress, and adjustment disorders

F50: Eating disorders

F54: Psychological and behavioral factors associated with disorders or diseases classified elsewhere

F79: Unspecified intellectual disabilities

F99: Mental disorder, not otherwise specified
